# Effect of Shallow Anterior Chamber on Postoperative Refractive Status in Patients of Age‐Related Cataract

**DOI:** 10.1155/joph/3854811

**Published:** 2026-04-17

**Authors:** Dongyan Xu, Shengnan Liu, Hui Zhang, Hui Dang, Wei Tang, Jing Wang

**Affiliations:** ^1^ Department of Ophthalmology, Jinan No.2 People’s Hospital, Jinan, Shandong, 250021, China

**Keywords:** age-related cataract, Barrett Universal II, hyperopic shift, intraocular lens calculation, refractive error, shallow anterior chamber

## Abstract

**Objective:**

To assess how shallow anterior chamber depth (ACD) affects refractive error (RE) after cataract surgery and compare modern intraocular lens (IOL) formulas in patients with normal axial length.

**Methods:**

This prospective study enrolled patients undergoing phacoemulsification with IOL implantation at Jinan No. 2 People’s Hospital (June 2023–December 2024). Patients were divided based on ACD into two groups. Biometrics were measured using IOLMaster 700, and IOL power was calculated with the Barrett Universal II (BUII) formula. Patients were reassessed at 1 and 3 months postoperatively. We analyzed correlations between RE and biometrics and compared BUII, Kane, EVO 2.0, and Hill‐RBF 3.0 formulas.

**Results:**

A total of 103 patients were divided into two groups: shallow (ACD < 2.5 mm, *n* = 57) and normal (ACD 2.5–3.5 mm, *n* = 46). Group Shallow had significantly greater RE (+0.451 ± 0.328 vs. −0.002 ± 0.277 D; *p* < 0.001) and greater postoperative ACD deepening (2.19 ± 0.26 mm vs. 1.43 ± 0.25 mm; *p* < 0.001) than group normal. Hyperopic shift correlated negatively with preoperative ACD and positively with ACD change (both *p* < 0.001). In shallow eyes, all formulas showed a hyperopic tendency, but BUII had the lowest mean absolute error (0.470 D, *p* < 0.001) and highest proportion of eyes within ±0.50 D (54.39%). In normal eyes, all formulas performed comparably well.

**Conclusion:**

Shallow ACD independently increases hyperopic shift risk. While all formulas are effective in normal eyes, BUII is superior in shallow chambers. Preoperative ACD and lens thickness assessment is crucial, and slight IOL power adjustment may optimize outcomes in shallow ACD cases.

## 1. Introduction

Cataract is the leading cause of blindness worldwide [1] and also a major contributor to low vision across all regions [2, 3]. In China, the prevalence of cataracts in people over 50 years old is as high as 27.45% [4, 5], and the disease burden rises with the aging population [6, 7]. Cataracts are irreversible, and phacoemulsification with intraocular lens (IOL) implantation is currently the only proven and effective treatment [8]. Cataract surgery historically focuses on restoring sight but has now evolved into precision refractive surgery, that is, tailoring visual outcomes through advanced diagnostics, IOL technology, and surgical techniques to help patients not just see but see well. As surgical precision and patient expectations continue to rise, minimizing postoperative refractive error (RE) and optimizing visual quality has become a key concern for both ophthalmologists and patients.

Existing studies confirm that the accuracy of preoperative refractive power prediction is a key determinant of postoperative visual quality [9]. The optimization of IOL formulas is one of the core components of preoperative refractive planning. IOL power calculation formulas differ in complexity, input parameters, and suitability for specific eye anatomies [10]. For traditional IOL formulas that rely on simplified assumptions and limited biometric inputs, prediction errors increase significantly in atypical situations, such as eyes with abnormal AL (< 22 mm or > 26 mm) or postrefractive eyes with altered keratometry (K) [11]. Newer formulas are designed to handle such challenging cases to avoid systematic errors [10]. For example, the Kane formula [12, 13] is a hybrid model rooted in theoretical optics that integrates regression analysis with artificial intelligence (AI). It uniquely calculates IOL power by first estimating the effective lens position (ELP) from parameters including axial length (AL), keratometry (K), anterior chamber depth (ACD), lens thickness (LT), and patient sex. The Hill‐RBF formula [14] is an AI‐based system that learns from a continuously expanding clinical database. Its self‐verifying and error‐correcting mechanism, which refines predictions using postoperative refractive outcomes, makes it an advanced and adaptive predictive tool. The EVO formula [15] is a thick‐lens model grounded in the emmetropization theory, which generates a personalized “emmetropization factor” for each eye. It uses AL, K, and ACD as core variables, with LT and central corneal thickness (CCT) serving as valuable optional inputs. The Barrett Universal II (BUII) formula [16] is a versatile and robust mainstay in modern cataract surgery. By incorporating AL, K, ACD, LT, and white‐to‐white (WTW) diameter while accounting for IOL principal plane shift, it delivers reliable ELP predictions with wide applicability and validated accuracy [17].

Norrby found that the largest source of postoperative RE in cataract surgery is the inaccurate prediction of ELP due to variability in ACD, which contributes to 35% of the total error, whereas AL measurement only contributes 17% [18]. It has been reported that a posterior ELP shift of 1 mm causes a hyperopic shift of +1.34 D [19], and accurate ELP prediction is thus needed to improve postoperative refractive results. Aging is associated with increased LT, which in turn reduces ACD [20, 21]. He et al. reported that in elderly Chinese (> 50 years old), the ACD is 2.49 ± 0.32 mm [22], and Zong et al. found that in healthy Chinese adults, ACD decreases at an average rate of 0.013 ± 0.005 mm per year. However, some conditions and anatomical traits are known to cause significantly shallower ACD, often below 2.2 mm or even 2.0 mm [23]. Patients with shallow ACD present unique challenges during cataract surgery and are more prone to postoperative REs. Although preoperative ACD is widely acknowledged as a key factor in ELP prediction error, most published studies typically analyze ACD in conjunction with AL. In comparison, the influence of LT on ACD shallowness has been largely overlooked. Therefore, this study aims to independently assess the impact of shallow ACD on postoperative REs following cataract surgery and to investigate whether it serves as a primary or independent contributing factor to such errors.

## 2. Methods

### 2.1. Patients

In this case–control study, we prospectively enrolled patients who underwent phacoemulsification combined with IOL implantation from June 2023 to December 2024 at Jinan No.2 People’s Hospital to treat age‐related cataracts. All patients had a normal AL, and they were divided into two groups based on their ACD [22], that is, Group Shallow (ACD < 2.5 mm) and Group Normal (2.5 mm ≤ ACD < 3.5 mm). Follow‐up data were collected at 1 month and 3 months postsurgery [24]. Patients were included if they were at least 55 years old, had age‐related cataracts, and satisfied the following:

• AL ranged in 22–24 mm and K ranged in 41–46 D
• No other fundus lesions were found on dilated eye exam
• No fundus treatment was administered before enrollment
• No intraoperative or postoperative complications occurred from the phacoemulsification combined with IOL implantation
• The target refraction was calculated using the BUII formula (16)
• Complete clinical data were available
 Patients were excluded if they had
• a history of eye trauma, refractive surgery, or intraocular surgery
• uveitis, corneal disease, fundus disease, glaucoma, or other eye diseases
• lens dislocation, capsule rupture, or zonular rupture during surgery
• high myopia (AL > 26 mm) or corneal astigmatism (> 1.0 D) in the operated eye
• autoimmune diseases, diabetes, or other major internal diseases
• incomplete clinical data.

This study strictly followed the principles of medical ethics and conformed to the guidelines of the Declaration of Helsinki. All research activities were reviewed and approved by the Ethics Committee of Jinan No. 2 People’s Hospital (Approval No. JNEYE20230104). The relevant risks were thoroughly explained to the patients, and all participating patients voluntarily provided their written informed consent.

### 2.2. Preoperative Exams

All patients underwent comprehensive preoperative ophthalmic evaluations, including assessments of unaided and corrected visual acuity, intraocular pressure measurement, slit‐lamp and fundus examination, corneal endothelial cell analysis, IOLMaster biometry, posterior segment optical coherence tomography (OCT), and ocular AB ultrasound.

Preoperative biometric evaluation of AL, K, and ACD was conducted using an IOLMaster 700 instrument (Carl Zeiss Meditec AG; Jena, Germany; system Version 1.90.33.04, *n*_*k*_ = 1.3375) by the same experienced ophthalmologist. Each eye was measured five times per parameter. The instrument automatically calculated the average value, and data were retained only when it signaled “OK” for internal quality check. To ensure reliable keratometry, eyes were excluded if the difference between the flat (K1) and steep (K2) meridians exceeded 1.00 D. To emphasize functional optics and reduce variability from corneal thickness, ACD was defined as the axial distance from the posterior corneal surface to the anterior surface of the crystalline lens [25, 26].

The measured biometric parameters were input into the online calculators of four formulas (BUII, Kane, EVO 2.0, and Hill‐RBF 3.0) to calculate the predicted IOL refractive power. A nominal A‐constant of 119.1, which is the optical value specified in the manufacturer’s instructions for the SY60WF IOL (Alcon; Fort Worth, TX, USA), was applied to all four formulas. For the BUII formula, this value corresponded to a lens factor of 1.936. The predicted equivalent spherical power (SE) corresponding to the IOL power recommended by each formula was recorded. The BUII calculation result was used as the actual IOL power for surgery.

### 2.3. Surgery and Follow‐Up

Preoperative evaluation included full physical and ocular examinations to rule out surgical contraindications. Beginning 3 days prior to surgery, patients instilled 0.5% levofloxacin eye drops (Cravit, Santen Pharmaceutical, Japan) four times daily to prevent infection. On the day of the procedure, 0.5% compound tropicamide drops (Mydrin‐P, Santen Pharmaceutical, Japan) were applied 30 min in advance to achieve adequate pupil dilation. All surgeries were performed by the same experienced physician using a Centurion phacoemulsification system (Alcon). Topical anesthesia was achieved with proparacaine hydrochloride eye drops (ALCAINE, Alcon). After a 2.2 mm main clear corneal incision was made at the 10 o’clock position, viscoelastic gel was injected into the anterior chamber. A 1.0 mm auxiliary incision was then created at 2 o’clock. A continuous curvilinear capsulorhexis (∼5.5 mm diameter) was performed, followed by hydrodissection to separate the lens cortex. The nucleus was then emulsified and removed, and residual cortical material was aspirated. Viscoelastic gel was then reinjected into the anterior chamber, and a monofocal aspheric one‐piece foldable posterior chamber IOL (SY60WF, Alcon) was gently implanted. The remaining viscoelastic gel was aspirated, and the primary incision was sealed to ensure watertight closure.

After the surgery, patients instilled 0.5% levofloxacin eye drops four times daily for 1 month and 1% prednisolone acetate eye drops (Pred Forte, Allergan; Dublin, Ireland) four times daily for 2 weeks. The refractive status and biometric parameters were re‐evaluated using the same IOLMaster 700 instrument one month and three months postoperatively. The change in ACD was calculated as ΔACD = ACD_followup_ − ACD_baseline_.

### 2.4. Refractive Outcome and Metrics

Three months postoperatively, patients underwent manifest refraction to determine the final refractive status. Initial objective measurements were obtained via automated refraction (NIDEK; Gamagori, Japan) and subsequently refined through subjective refraction. The actual refractive outcome was quantified as the equivalent spherical value, that is, the sum of the measured SE and half the cylindrical correction. RE [27] was defined as the numerical difference between the postoperative refractive value and the preoperative target (reserved refractive power), with negative RE values indicating myopic shift and positive values indicating hyperopic shift [28]. This approach allowed standardized quantification of refractive outcomes and directional bias relative to preoperative expectations.

The accuracy metrics for evaluating the four IOL formulas included RE, mean RE, mean absolute RE (MAE), median absolute error (MedAE), and the percentage of RE within the ±0.25, ±0.50, and ±1.00 D intervals. Because RE may cancel each other upon summation, we used MAE as the standard for statistical analysis. For patients in Group Shallow, we also analyzed the correlation between the RE obtained from the BUII formula and preoperative/postoperative biometric parameters.

### 2.5. Statistical Analysis

Statistical analyses were performed using SPSS Version 27.0 (SPSS Inc., Chicago, IL, USA). The normality of continuous variables was assessed using the Shapiro–Wilk test. Data conforming to a normal distribution are presented as the mean ± standard deviation (SD), while non‐normally distributed data are reported as the median and interquartile range (IQR).

For group comparisons, MAE was analyzed using nonparametric tests. Differences in MAE between groups stratified by ACD were evaluated by the Wilcoxon rank‐sum test, and differences in MAE among various IOL formulas were compared by the Friedman test. The percentages of eyes within a specific RE interval were compared by Cochran’s *Q* test.

The relationship between postoperative RE and both preoperative ACD and postoperative ACD change was evaluated using Pearson’s linear correlation analysis. The strength of correlation was interpreted as follows: |r| < 0.30, negligible; 0.30 ≤ |r| < 0.50, low; 0.50 ≤ |r| < 0.80, moderate; and |r| ≥ 0.80, high. A two‐tailed *p*‐value of < 0.05 was considered statistically significant for all tests except the correlation analysis, for which a threshold of *p* < 0.01 was applied.

## 3. Results

Table 1 summarizes the baseline characteristics of the enrolled patients. A total of 103 patients (103 eyes) were included, and each patient contributed one affected eye. The entire cohort included 49 male and 54 female participants, with age ranging from 56 to 85 years (mean 68.78 ± 5.95 years). Group Shallow included 57 patients (57 eyes), and Group Normal included 46 patients (46 eyes). There were no significant differences in baseline parameters between the two groups (all *p* > 0.05), including age, sex distribution, AL, and K.

**TABLE 1:** Patient characteristics.

Characteristics	Normal (*n* = 46)	Shallow (*n* = 57)	*t*	*χ* ^2^	*p*
Age (years)^§^	68.43 ± 4.89	69.05 ± 6.71	−0.522		0.603
Sex (M/F)	22/24	27/30		0.321	0.768
Eyes	46	57			
Axial length (AL, mm)^§^	22.82 ± 0.49	22.72 ± 0.51	0.986		0.327
Keratometry (K, D)^§^	44.21 ± 1.64	44.25 ± 1.32	−0.107		0.915

^§^Expressed as mean ± SD.

Table 2 shows that 3 months after the surgery, the increase of ACD was 1.43 ± 0.25 mm in Group Normal and 2.19 ± 0.26 mm in Group Shallow, and the difference in deepening was significant (*t* = −15.05, *p* < 0.001). Group Shallow had significantly greater MAE compared to Group Normal (0.470 vs. 0.224 D; *p* < 0.001). The significantly greater RE in Group Shallow compared to Group Normal (+0.451 ± 0.328 vs. −0.002 ± 0.277 D; *t* = −7.454, *p* < 0.001) matched a hyperopic refractive shift. Across all eyes, unaided postoperative visual acuity ranged from 0.6 to 1.0, and corrected visual acuity ranged from 0.8 to 1.0.

**TABLE 2:** Comparison of biometric and refractive measures before and after surgery.

	Normal	Shallow	*t/z*	**p**

Number of eyes	46	57		
Preoperative				
LT_0_ (mm)^§^	4.38 ± 0.37	5.17 ± 0.34	11.15	< 0.001
ACD_0_ (mm)^§^	3.02 ± 0.25	2.16 ± 0.21	18.438	< 0.001
One month postoperative				
LT_1_ (mm)^§^	0.83 ± 0.14	1.01 ± 1.09	1.11	0.270
ACD_1_ (mm)^§^	4.44 ± 0.19	4.34 ± 0.15	−2.79	0.006
ΔACD_1_ (mm)^§^	1.42 ± 0.25	2.19 ± 0.25	15.11	< 0.001
RE_1_ (D)^§^	−0.006 ± 0.268	+0.446 ± 0.327	7.54	< 0.001
MAE_1_ (D)^§^	0.223	0.471	−4.631	< 0.001
Three months postoperative				
LT_3_ (mm)^§^	0.83 ± 0.14	1.01 ± 1.09	1.11	0.270
ACD_3_ (mm)^§^	4.45 ± 0.19	4.35 ± 0.16	−2.87	0.005
ΔACD_3_ (mm)^§^	1.43 ± 0.25	2.19 ± 0.26	−15.05	< 0.001
RE_3_ (D)^§^	−0.002 ± 0.277	+0.451 ± 0.328	−7.454	< 0.001
MAE_3_ (D)^§^	0.224	0.470	−4.177	< 0.001

*Note:* ΔACD, increase of ACD; RE was indistinguishable at 1 month and 3 months postoperatively (*p* = 0.94). Subscripts 0, 1, and 3 denote data measured preoperatively, at 1 month postoperatively, and at 3 months postoperatively, respectively.

Abbreviations: ACD, anterior chamber depth; LT, lens thickness; MAE, mean absolute error; RE, refractive error.

^§^Expressed as mean ± SD.

**FIGURE 1:**
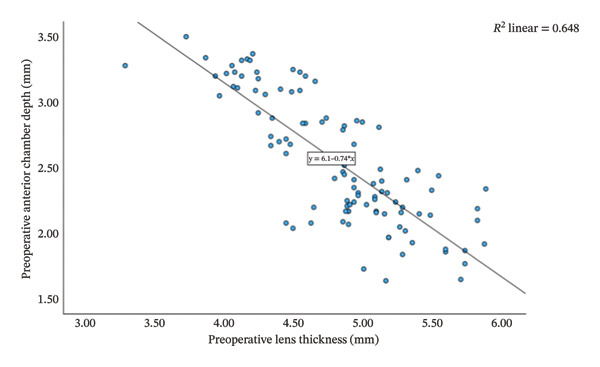
Correlations between preoperative lens thickness and preoperative anterior chamber depth in all cataract patients. ACD_0_ = 6.1−0.74LT_0_.

**FIGURE 4:**
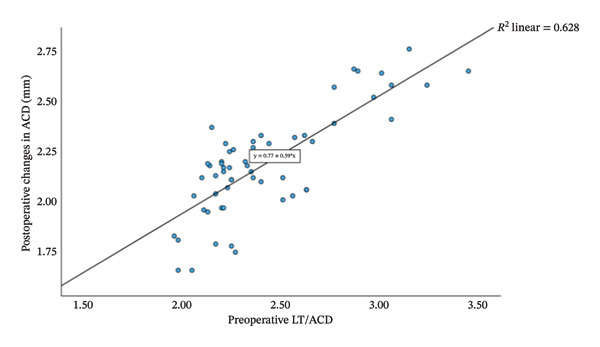
Correlations between preoperative LT/ACD and postoperative change in anterior chamber depth (measured at 3 months) for cataract patients with shallow anterior chamber. ΔACD_3_ = 0.77 + 0.59LT_0_/ACD_0_.

**FIGURE 5:**
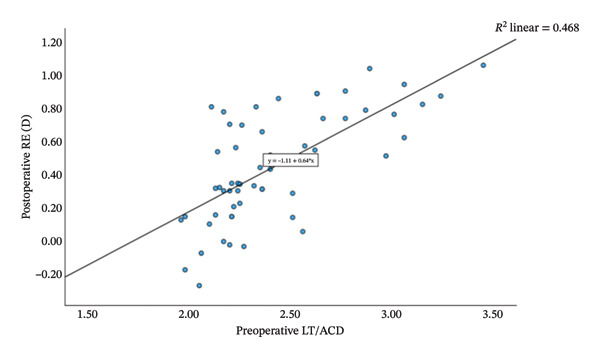
Correlations between preoperative LT/ACD and postoperative refractive error (measured at 3 months) for cataract patients with shallow anterior chamber. RE_3_ = −1.11 + 0.64LT_0_/ACD_0_.

Group Shallow had significantly greater preoperative LT than Group Normal (*t* = 11.15, *p* < 0.001, Table 2). Furthermore, an increase in LT was significantly and negatively correlated with the reduction in ACD (*r* = −0.81, *p* < 0.001; Figure 1). That is, a greater preoperative LT could lead to a more pronounced increase in ACD after the surgery, which would exacerbate RE. The two groups did not differ significantly in postoperative LT (*p* = 0.270 for both time points, Table 2). For Group Normal, RE was not correlated significantly with either preoperative ACD (*r* = 0.255, *p* > 0.05) or postoperative ACD change (*r* = −0.187, *p* > 0.05). In contrast, for Group Shallow, RE had a moderate negative correlation with preoperative ACD (1 month, *r* = −0.730, *p* < 0.01; 3 months, *r* = −0.734, *p* < 0.01; Figures 2 and 3) and a moderate positive correlation with postoperative ACD change (1 month, *r* = 0.683, *p* < 0.01; 3 months, *r* = 0.684, *p* < 0.01; Figures 2 and 3). In addition, the preoperative LT/ACD ratio was significantly positively correlated with two metrics measured at 3 months postoperative, that is, postoperative ACD change (*r* = 0.792, *p* < 0.001; Figure 4) and postoperative RE (*r* = 0.684, *p* < 0.001; Figure 5). That is, when the LT/ACD ratio is higher (i.e., shallower preoperative anterior chamber and thicker lens), the postoperative ACD change is greater and the RE increases (i.e., hyperopic shift).

FIGURE 2Correlations between refractive error at 1 month postoperatively and (a) preoperative anterior chamber depth and (b) postoperative change in anterior chamber depth for cataract patients with shallow anterior chamber. ACD_0_ = 2.37−0.47RE_1_, ΔACD_1_ = 1.95 + 0.54RE_1_.

**Figure:**
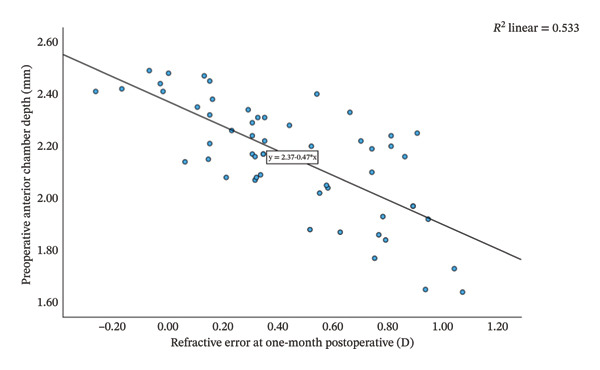
(a)

**Figure:**
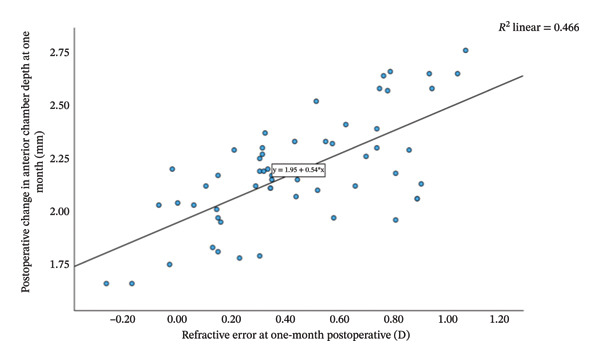
(b)

FIGURE 3Correlations between refractive error at 3 months postoperatively and (a) preoperative anterior chamber depth and (b) postoperative change in anterior chamber depth for cataract patients with shallow anterior chamber. ACD_0_ = 2.37−0.47RE_3_, ΔACD_3_ = 1.95 + 0.54RE_3_.

**Figure:**
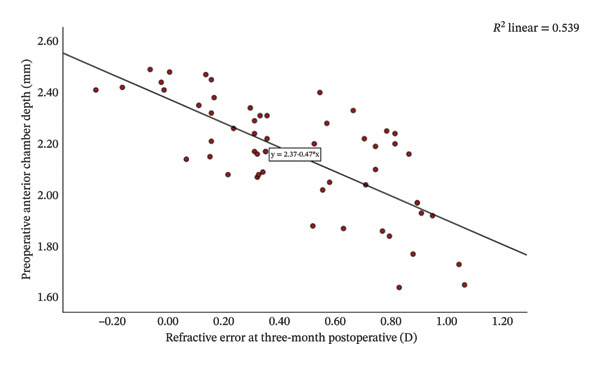
(a)

**Figure:**
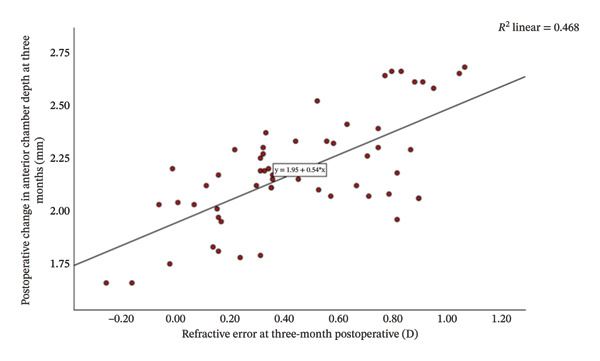
(b)

**FIGURE 6:**
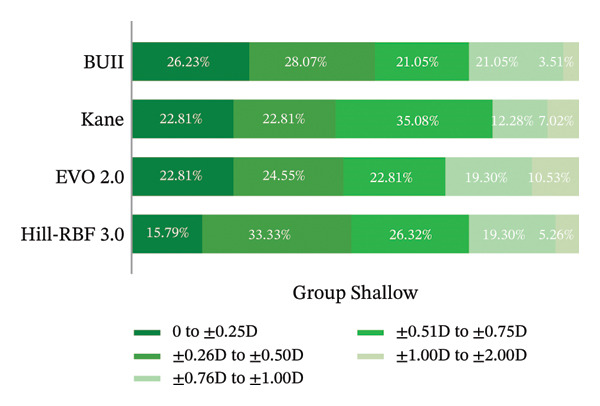
Refractive outcomes of Group Shallow measured at 3 months postoperatively.

**FIGURE 7:**
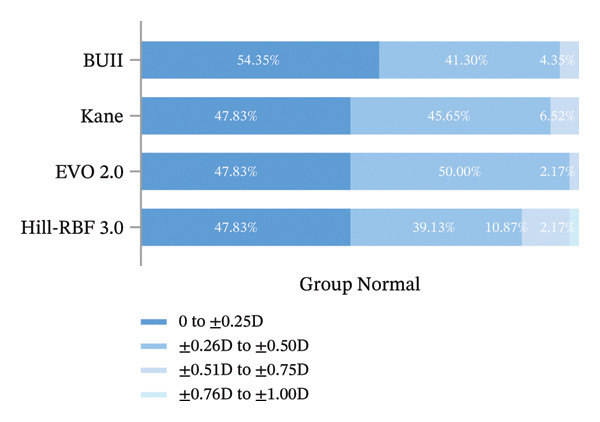
Refractive outcomes of Group Normal measured at 3 months postoperatively.

The analysis of IOL formula prediction accuracy revealed a pronounced divergence in performance between eyes with shallow and normal ACD. For Group Shallow, all four formulas showed a tendency toward hyperopic outcomes, but their prediction accuracy differed significantly. BUII gave the lowest mean RE (+0.451 ± 0.328 D, *p* < 0.001), the lowest MAE (0.470 D, *p* < 0.001), and the lowest MedAE (0.350 D). It also yielded the highest proportion of eyes meeting desirable refractive targets, with 26.32% within ±0.25 D and 54.39% within ±0.50 D (Table 3, Figure 6), although these distributional advantages were not statistically significant. In contrast, for Group Normal, all formulas achieved consistently excellent outcomes, and the mean RE was statistically indistinguishable (*p* = 0.958). Although BUII again attained the best metrics numerically (MAE 0.224 D, *p* = 0.063; MedAE 0.188 D), its difference from other formulas was not significant. For all formulas, 100% of the eyes achieved a refractive outcome within ±1.00 D, and most attained ±0.50 D (BUII, 95.65%; Kane, 93.48%; EVO 2.0, 97.83%; Hill‐RBF 3.0, 86.96%; Table 3, Figure 7).

**TABLE 3:** Comparison of prediction errors of IOL formulas.

Prediction error^§^	Group^¶^	BUII	Kane	EVO 2.0	Hill‐RBF 3.0	*p*
Refractive error (D)	Shallow	+0.451 ± 0.328	+0.499 ± 0.365	+0.553 ± 0.392	+0.510 ± 0.346	< 0.001
	Normal	−0.002 ± 0.277	+0.023 ± 0.296	−0.010 ± 0.287	+0.003 ± 0.330	0.958

Breakdown of refractive error						

0 to ±0.25 D	Shallow	26.32%	22.81%	22.81%	15.79%	0.123
	Normal	54.35%	47.83%	47.83%	47.83%	0.717

±0.26 to ±0.50 D	Shallow	28.07%	22.81%	24.55%	33.33%	0.334
	Normal	41.30%	45.65%	50.00%	39.13%	0.523

±0.51 to ±0.75 D	Shallow	21.05%	35.08%	22.81%	26.32%	0.108
	Normal	4.35%	6.52%	2.17%	10.87%	0.232

±0.76 to ±1.00 D	Shallow	21.05%	12.28%	19.30%	19.30%	0.422
	Normal	0.00%	0.00%	0.00%	2.17%	1.000

±1.01 to ±2.00 D	Shallow	3.51%	7.02%	10.53%	5.26%	0.233
	Normal	0.00%	0.00%	0.00%	0.00%	/

*Subtotals*						

within ±0.50 D	Shallow	54.39%	45.61%	47.37%	49.12%	0.397
	Normal	95.65%	93.48%	97.83%	86.96%	0.104

within ±0.75 D	Shallow	75.44%	80.70%	70.18%	75.44%	0.153
	Normal	100.00%	100.00%	100.00%	97.83%	1.000

within ±1.00 D	Shallow	96.49%	92.98%	89.47%	94.74%	0.233
	Normal	100.00%	100.00%	100.00%	100.00%	/

Mean absolute error (D)	Shallow	0.470	0.533	0.569	0.533	< 0.001
	Normal	0.224	0.253	0.247	0.265	0.063

Median absolute error (D)	Shallow	0.350 [0.230, 0.740]	0.510 [0.335, 0.740]	0.560 [0.325, 0.830]	0.510 [0.330, 0.705]	/
	Normal	0.188 [0.070, 0.370]	0.263 [0.120, 0.370]	0.268 [0.120, 0.350]	0.290 [0.110, 0.390]	/

Maximum absolute error (D)	Shallow	1.060	1.320	1.660	1.470	/
	Normal	0.530	0.540	0.540	0.820	/

*Note:* percentage of eyes, or median [P25, P75]. All values are calculated based on the data collected at 3 months postoperatively.

^§^Expresses as mean ± standard deviation.

^¶^Group Shallow, *n* = 57; Group Normal, *n* = 46.

## 4. Discussion

Cataract is a leading cause of vision loss, and its prevalence increases with age. Phacoemulsification with IOL implantation is now considered the standard of care and remains the most widely adopted treatment. Advancements in biometric instruments, surgical techniques, and functional IOL have transformed cataract surgery from sight‐restoring to refractive surgery [29]. Patients seek not just to regain vision postoperatively but to experience clear, comfortable, and enduring vision. The rising expectation of visual quality requires improving surgical refractive outcomes [30].

The accuracy of postoperative refractive power, which is a key determinant of patient satisfaction, depends on reliable ocular biometric inputs (e.g., AL, ACD, and K), appropriate selection of IOL calculation formula, and precise ELP prediction [31]. If biometric parameters are reliably measured, ELP prediction becomes the dominant variable influencing postoperative RE. ELP refers to the predicted optical position of the IOL, defined as the distance from the posterior corneal surface to the IOL’s principal plane along the visual axis. It cannot be measured directly prior to surgery and is instead inferred from biometric parameters such as AL, ACD, K, and LT. A forward shift in ELP places the IOL closer to the cornea, increasing the eye’s overall optical power and resulting in a myopic RE; conversely, a backward shift in ELP reduces the eye’s optical power, leading to a hyperopic error [32, 33].

Accurate preoperative measurements are critical for achieving optimal refractive outcomes after cataract surgery. The IOLMaster is a noncontact optical biometer that enhances measurement accuracy and reliability by utilizing swept‐source OCT [34]. This non‐invasive technology eliminates measurement errors caused by corneal indentation, a common issue with traditional contact ultrasound methods. By measuring the distance from the tear film to the macular region with high precision and repeatability (up to 0.01 mm), the IOLMaster provides superior biometric data unaffected by intermediary intraocular structures [35]. In the present work, all biometric assessments were performed using IOLMaster 700. To ensure measurement reliability, patients with severe media opacity or poor fixation were excluded.

The IOLMaster 700 supports various third‐, fourth‐, and fifth‐generation formulas for IOL power calculation. For eyes with normal AL, K, and ACD, calculation formulas including SRK/T, Hoffer *Q*, Haigis, Holladay 2, and BUII all show high predictive accuracy and consistency [36]. However, eyes with atypical biometric features (such as shallow ACD or extreme values AL) often undergo more pronounced anatomical changes following cataract surgery. These changes are less reliably predicted by standard preoperative measurements, which increases the difficulty of accurate IOL positioning and the likelihood of postoperative RE. In this study, although both groups had normal AL and K, their ACD differed significantly (Group Normal, 3.02 ± 0.25 mm; Group Shallow, 2.16 ± 0.21 mm; *p* < 0.001). The SRK/T formula is a third‐generation method that overlooks ACD and uses only AL and K in estimating ELP [37], and it is less reliable in dealing with eyes with shallow chambers. Hoffer *Q*, also a third‐generation formula, relies on AL and K along with a personalized ACD constant (pACD), but it may still misestimate ELP in eyes with shallow ACD, risking myopic outcomes [38]. The fourth‐generation formula Haigis, which incorporates AL, K, and ACD through three constants (*a*_0_,  *a*_1_,  *a*_2_), offers improved accuracy but still shows mild myopic bias in long eyes [39]. Holladay 2 is a multivariable, fourth‐generation formula that incorporates seven biometric parameters, including ACD, but it shows diminished accuracy in eyes with shallow ACD, often producing hyperopic outcomes due to underestimation of ELP [40].

Building on the critique of traditional theoretical formulas, modern IOL calculations have advanced through two principal approaches: data‐driven AI systems and sophisticated ray‐tracing optics. AI‐based formulas, such as Hill‐RBF, Kane, and EVO, leverage large clinical datasets and adaptive learning algorithms to refine predictions. Rather than relying on fixed theoretical eye models, these formulas identify complex, nonlinear relationships between biometric parameters and postoperative refraction, continually improving their accuracy as their databases expand. In contrast, the BUII formula, which employs Snell’s law‐based ray tracing to simulate light propagation through the eye, represents a complementary advancement grounded in Gaussian optics [16]. It achieves a more individualized and physically realistic estimation of ELP by integrating multiple anatomical parameters, including AL, ACD, K, LT, and WTW, and accounting for how the IOL’s optical principal plane shifts with lens power [41]. This combination of optical modeling has consistently demonstrated high predictive accuracy [42, 43], particularly in eyes with challenging anatomy such as shallow anterior chambers [17, 44]. Our findings demonstrate that in eyes with shallow anterior chambers, all four evaluated formulas exhibited a mean hyperopic prediction error, and the magnitude of the error fell in the order of BUII < Kane < Hill‐RBF 3.0 < EVO 2.0 (*p* < 0.001). Although the differences in the proportion of eyes within specific RE ranges did not reach statistical significance, BUII consistently yielded the highest percentage of outcomes within ±0.25 D, ±0.50 D, and ±1.00 D intervals. This pattern indicates a superior reliability profile for BUII in this anatomically challenging group, which aligns with prior large‐scale analyses of Melles et al., who reported superior performance of BUII in eyes with shallow anterior chambers [17].

The refractive impact of shallow ACD remains an ongoing research topic. Huang et al. [45] reported that ACD increases significantly after cataract surgery, and the degree of deepening is inversely correlated to preoperative ACD (i.e., shallower chambers experience greater postoperative change). Bilak et al. [46] found that individuals with deeper preoperative ACD maintain a decreased lead postoperatively, as a 1.0 mm difference before surgery translates to only a 0.6–0.7 mm difference after surgery. Dong et al. [47] found that shallow ACD gives rise to postoperative hyperopia deviation and highlighted the importance of preoperative ACD in forecasting refractive status after cataract surgery. In our study, at 1‐month follow‐up, patients in Group Shallow exhibited a greater ACD increase (2.19 ± 0.25 mm) with a notable hyperopic shift (+0.446 ± 0.327 D), whereas those in Group Normal had a smaller ACD deepening (1.42 ± 0.25 mm) and a slight myopic shift (−0.006 ± 0.268 D). The degree of hyperopic shift correlated significant negatively with preoperative ACD (*r* = −0.730, *p* < 0.01) and significantly positively with postoperative ACD change (*r* = 0.683, *p* < 0.01). The results at 3‐month follow‐up were identical (Figure 2). That is, shallower preoperative ACD was associated with greater postoperative deepening of the anterior chamber and more pronounced hyperopic shift.

Various other studies also demonstrate that shallower preoperative ACD predicts greater postoperative deepening and a tendency toward hyperopic RE. Yang et al. [48] disclosed that in age‐related cataract patients, shallower preoperative ACD is significantly associated with greater postoperative hyperopic shift, and refractive deviation increases as ACD decreases. Similarly, Sun et al. [49] reported that in patients with age‐related cataract and shallow anterior chambers, greater postoperative hyperopic shift after phacoemulsification is associated with shallower preoperative ACD and larger postoperative increases in ACD. A few mechanisms contribute to such a phenomenon. For example, patients with shallow preoperative ACD often exhibit anatomical features such as a thick lens‐iris diaphragm, increased LT, or zonular laxity, which predispose the eye to greater anterior segment instability following lens extraction. Removal of the crystalline lens reduces posterior capsular support, allowing the vitreous to shift forward and intraocular pressure to drop transiently, both of which can contribute to axial shortening and postoperative hyperopic shift. Additionally, the mechanical expansion of the globe by IOL haptics may increase the transverse diameter and subtly shorten the visual axis, further exacerbating hyperopic outcomes, particularly in short eyes. Besides, shallow ACD is also frequently associated with a disproportionate ACD/AL ratio, which impairs the accuracy of ELP prediction in biometric formulas and increases the likelihood of RE [50]. Finally, preoperative macular thickening, whether due to edema or retinal pathology, can inflate AL values, leading to the underestimation of the required IOL power and contributing to postoperative hyperopia [51].

However, Ning et al. [52] found that hyperopia happens to those with smaller ACD changes instead, and eyes with greater postoperative ACD deepening (> 1.65 mm) tend to experience a myopic shift, and they attributed the observation to the posterior displacement of the IOL with deeper chambers. This discrepant finding may have stemmed from methodological differences. They included patients with a broad range of AL (including extreme values at < 22 mm and > 26 mm) and applied different IOL formulas (Hoffer *Q*, SRK/T, and Haigis) based on the AL. The heterogeneity in ocular anatomy and formula selection may have introduced variability in ELP predictions, potentially reducing the reliability of their refractive outcome analysis. Hence, the link between shallow anterior chambers and postoperative refractive shift remains inconclusive and warrants further investigation through large‐scale clinical studies. Clinically, surgeons should be alert to the risk that, in patients with shallow preoperative ACD, postoperative deepening of the anterior chamber may lead to hyperopic shift. Careful biometric assessment and clear patient counseling are key to achieving optimal visual outcomes. Adjusting IOL power selection during preoperative planning and setting realistic refractive expectations are essential.

This work has several limitations. First, the sample size was not determined through formal power calculation prior to study initiation. The current cohort reflects the available patient population during the study period and is intended to generate initial observations to inform future research. Larger, adequately powered studies are needed to validate the present findings. Second, the study evaluated only a single type of IOL (a monofocal aspheric model), and the results may not be generalizable to other IOL designs, particularly functional or multifocal lenses. Separate investigations are needed to assess how different IOL types perform in eyes with complicated ACD. Third, while the final refractive outcome was determined by subjective manifest refraction, the process was initiated by automated refraction. Although this two‐step process is standard in clinical practice, it could introduce minimal variability compared to a protocol relying solely on meticulous manual refraction. Fourth, while the influence of surgical astigmatism was considered by selecting time points with relatively stable refractive status (1 and 3 months postoperatively), individual variability in healing and corneal remodeling may still introduce confounding effects.

## 5. Conclusions

We found that patients with age‐related cataract are predisposed to a postoperative hyperopic shift if they have a shallow ACD, even when their AL is normal. The refractive inaccuracy is driven by a significant postoperative deepening of the anterior chamber, which is inversely correlated with preoperative ACD and positively correlated with LT. The predictive accuracy of four modern IOL formulas (BUII, Kane, EVO 2.0, Hill‐RBF 3.0) was assessed. They performed excellently and comparably in eyes with normal ACD, but BUII gave the highest predictive accuracy in eyes with shallow chambers.

The key clinical insight is that preoperative ACD and LT are critical, independent factors for refractive prediction. Surgeons should be aware that a shallower preoperative ACD and a greater LT elevate the risk of an unexpected hyperopic outcome. In such cases, relying on the BUII formula for calculation and considering a slight intentional targeting of myopia (i.e., selecting a slightly higher IOL power) may help neutralize the hyperopic shift and optimize the final refractive result.

## Author Contributions

Conceptualization: Dongyan Xu and Jing Wang; methodology: Dongyan Xu; software, Dongyan Xu and Shengnan Liu; validation: Dongyan Xu, Shengnan Liu, Hui Zhang, Hui Dang, Wei Tang, and Jing Wang; formal analysis: Dongyan Xu; investigation: Dongyan Xu and Shengnan Liu; data curation: Dongyan Xu, Shengnan Liu, Hui Zhang, Hui Dang, and Wei Tang; writing–original draft preparation: Dongyan Xu; writing–review and editing: Dongyan Xu and Jing Wang.

## Funding

This study was supported by The Jinan Municipal Health Commission Science and Technology Development Plan Project, 2023‐1‐31.

Open access funding was enabled and organized by Projekt DEAL.

## Ethics Statement

This study strictly followed the principles of medical ethics and conformed to the guidelines of the Declaration of Helsinki. All research activities were reviewed and approved by the Ethics Committee of Jinan No. 2 People’s Hospital (Approval No. JNEYE20230104). The relevant risks were thoroughly explained to the patients, and all participating patients voluntarily provided their written informed consent.

## Conflicts of Interest

The authors declare no conflicts of interest.

## Data Availability

The datasets used and analyzed during the current study are available from the corresponding author on request.

## References

[1] World Health Organization WHO , Blindness and Vision Impairment, 2023, https://www.who.int/news-room/fact-sheets/detail/blindness-and-visual-impairment.

[2] GBD 2019 Blindness and Vision Impairment Collaborators , Causes of Blindness and Vision Impairment in 2020 and Trends over 30 years, and Prevalence of Avoidable Blindness in Relation to Vision 2020: THE Right to Sight: An Analysis for the Global Burden of Disease Study, Lancet Global Health. (2021) 9, no. 2, e144–e160, 10.1016/s2214-109x(20)30489-7. 33275949 PMC7820391

[3] Lin L. , Liang Y. , Jiang G. et al., Global, Regional, and National Burden of Cataract: A Comprehensive Analysis and Projections from 1990 to 2021, PLoS One. (2025) 20, no. 6, 10.1371/journal.pone.0326263. PMC12185006 40549768

[4] Du Y. F. , Liu H. R. , Zhang Y. et al., Prevalence of Cataract and Cataract Surgery in Urban and Rural Chinese Populations Over 50 Years Old: A Systematic Review and Meta-Analysis, International Journal of Ophthalmology. (2022) 15, no. 1, 141–149, 10.18240/ijo.2022.01.21. 35047369 PMC8720354

[5] Xu T. , Wang B. , Liu H. et al., Prevalence and Causes of Vision Loss in China From 1990 to 2019: Findings From the Global Burden of Disease Study 2019, Lancet Public Health. (2020) 5, no. 12, e682–e691, 10.1016/s2468-2667(20)30254-1. 33271081

[6] Fang R. , Yue P. L. , Ding X. F. et al., The Burden of Vision Loss Due to Cataract in China: Findings From the Global Burden of Disease Study 2019, Eye. (2024) 38, no. 5, 885–892, 10.1038/s41433-023-02798-0. 37853108 PMC10965953

[7] Gao Y. , Liu J. , Zhou W. , Tian J. , Wang Q. , and Zhou L. , Exploring Factors Influencing Visual Disability in the Elderly Population of China: A Nested Case-Control Investigation, Journal of global health. (2023) 13, 10.7189/jogh.13.04142. PMC10644848 37962345

[8] Iroku-Malize T. and Kirsch S. , Eye Conditions in Older Adults: Cataracts, FP essentials. (2016) 445, 17–23. 27348528

[9] Xu D. Y. and Wang J. , Factors Affecting the Refractive Error After Cataract Surgery, International Ophthalmology. (2025) 45, no. 1, 10.1007/s10792-025-03543-0. 40319199

[10] Stopyra W. , Langenbucher A. , and Grzybowski A. , Intraocular Lens Power Calculation Formulas-A Systematic Review, Ophthalmology and therapy. (2023) 12, no. 6, 2881–2902, 10.1007/s40123-023-00799-6. 37698825 PMC10640516

[11] Rossip M. G. , Hastings J. , Burwinkel H. et al., Relative Behavior of Modern Intraocular Lens Power Calculation Formulas Across a Realistic Range of Biometry Values, Clinical Ophthalmology. (2025) 19, 2037–2045, 10.2147/opth.s529208. 40612333 PMC12223413

[12] Zhu K. K. , Wang X. , and Mu H. M. , Analyze the Effect of Keratometry on the Calculation Accuracy of Intraocular Lens Diopter in Patients With Normal Axial Cataract, International Eye Science. (2022) 22, no. 4, 633–636, 10.3980/j.issn.1672-5123.2022.4.21.

[13] Yang S. , Shao J. , and Zhang J. , Application of Artificial Intelligence in Intraocular Lens Power Calculation, International Eye Science. (2022) 22, no. 5, 716–720, 10.3980/j.issn.1672-5123.2022.5.04.

[14] Tsessler M. , Cohen S. , Wang L. , Koch D. D. , Zadok D. , and Abulafia A. , Evaluating the Prediction Accuracy of the Hill-RBF 3.0 Formula Using a Heteroscedastic Statistical Method, Journal of Cataract & Refractive Surgery. (2022) 48, no. 1, 37–43, 10.1097/j.jcrs.0000000000000702. 34016821

[15] Zhang C. , Ye Z. , and Li Z. , Advances in Intraocular Lens Power Calculation Formulas in High Myopia, Chinese Journal of Experimental Ophthalmology. (2022) 40, no. 5, 466–469, 10.3760/cma.j.cn115989-20200512-00341.

[16] Jennings E. and Hall B. , A Retrospective Study of Visual Outcomes When Using a Cloud-Based Surgical Planning Platform and the Barrett Universal Ii Formula, Clinical Ophthalmology. (2024) 18, 2605–2609, 10.2147/opth.s481797. 39309686 PMC11416102

[17] Melles R. B. , Holladay J. T. , and Chang W. J. , Accuracy of Intraocular Lens Calculation Formulas, Ophthalmology. (2018) 125, no. 2, 169–178, 10.1016/j.ophtha.2017.08.027, 2-s2.0-85029764746. 28951074

[18] Norrby S. , Sources of Error in Intraocular Lens Power Calculation, Journal of Cataract & Refractive Surgery. (2008) 34, no. 3, 368–376, 10.1016/j.jcrs.2007.10.031, 2-s2.0-41949113224. 18299059

[19] Jonas J. B. , Nangia V. , Gupta R. et al., Anterior Chamber Depth and Its Associations With Ocular and General Parameters in Adults, Clinical and Experimental Ophthalmology. (2012) 40, no. 6, 550–556, 10.1111/j.1442-9071.2011.02748.x, 2-s2.0-84865179758. 22171546

[20] Lei Q. , Tu H. , Feng X. , Ortega-Usobiaga J. , Cao D. , and Wang Y. , Distribution of Ocular Biometric Parameters and Optimal Model of Anterior Chamber Depth Regression in 28,709 Adult Cataract Patients in China Using Swept-Source Optical Biometry, BMC Ophthalmology. (2021) 21, no. 1, 10.1186/s12886-021-01932-4. PMC8045194 33849464

[21] Meng J. , Wei L. , He W. , Qi J. , Lu Y. , and Zhu X. , Lens Thickness and Associated Ocular Biometric Factors Among Cataract Patients in Shanghai, Eye and vision (London, England). (2021) 8, no. 1, 10.1186/s40662-021-00245-3. PMC8165789 34053465

[22] He M. , Huang W. , Zheng Y. , Alsbirk P. H. , and Foster P. J. , Anterior Chamber Depth in Elderly Chinese: The Liwan Eye Study, Ophthalmology. (2008) 115, no. 8, 1286–1290, 10.1016/j.ophtha.2007.12.003, 2-s2.0-48149091424. 18471885

[23] Jarstad J. S. , Mina H. , and Midathala G. , Pardianto G. and Tassignon M-J. , Cataract Surgery in Challenging Cases: A Review, Innovation in Cataract Surgery, 2024, Springer Nature Singapore, Singapore, 153–159.

[24] Charlesworth E. , Alderson A. J. , de Juan V. , and Elliott D. B. , When is Refraction Stable Following Routine Cataract Surgery? A Systematic Review and Meta-Analysis, Ophthalmic and Physiological Optics. (2020) 40, no. 5, 531–539, 10.1111/opo.12719. 32696501

[25] Mao Y. , Li J. , Qin Y. et al., Association of Refractive Outcome With Postoperative Anterior Chamber Depth Measured with 3 Optical Biometers, International Ophthalmology. (2024) 44, no. 1, 10.1007/s10792-024-02995-0. 38345699

[26] Findl O. , Hirnschall N. , and Kronschläger M. , Aramberri J. , Hoffer K. J. , Olsen T. , Savini G. , and Shammas H. J. , Anterior Chamber Depth and Iol Calculations, Intraocular Lens Calculations, 2024, Springer International Publishing, Cham, 537–550.

[27] Shajari M. , Sonntag R. , Niermann T. et al., Determining and Comparing the Effective Lens Position and Refractive Outcome of a Novel Rhexis-Fixated Lens to Established Lens Designs, American Journal of Ophthalmology. (2020) 213, 62–68, 10.1016/j.ajo.2020.01.009. 31953058

[28] Song X. , Chen B. , Wang Y. , Li J. , and Xu Y. , 23-Gague Anterior Vitrectomy During Phacoemulsification for Cataract With Low Corneal Endothelial Cell Density and Shallow Anterior Chamber, Recent Advances in Ophthalmology. (2016) 36, no. 11, 1058–1060, 10.13389/j.cnki.rao.2016.0282.

[29] Sharma A. and Batra A. , Evaluation of Scheimpflug Imaging System as an Added Tool in Improving the Accuracy of Reference Marking (As Compared to the Slit Lamp Marking System) for Toric Intraocular Lens Implantation, Indian Journal of Ophthalmology. (2020) 68, no. 4, 583–586, 10.4103/ijo.IJO_1253_19. 32174573 PMC7210844

[30] Zhou J. , Zhan X. , Huo Y. , and Ye J. , Visual Effects of Trifocal Intraocular Lens Implantation in Cataract Patients With Different Refractive States, BMC Ophthalmology. (2025) 25, no. 1, 10.1186/s12886-025-03963-7. PMC11905677 40075276

[31] Lee N. S. and Ong K. , Factors Contributing to Long-Term Refractive Error After Cataract Surgery, International Ophthalmology. (2023) 43, no. 7, 2335–2340, 10.1007/s10792-022-02630-w. 36592262

[32] Wu P. , Sun Y. , Peng H. , Liu Z. , Wen Y. , and Chen M. , Influence of Ocular Biometric Parameters Such as Effective Lens Position, Keratometry, and Axial Length on Near Add Power of Multifocal Intraocular Lens, Journal of Cataract & Refractive Surgery. (2022) 48, no. 11, 1331–1334, 10.1097/j.jcrs.0000000000000947. 35405733

[33] Tan J. , Qin Y. , Wang C. , and Ye J. , Influences of Different Reserved Diopters During Cataract Extraction Combined With Tecnis Symfony Intraocular Lens Implantation on Visual Quality of Presbyopia Correction, Chinese Journal of Experimental Ophthalmology. (2019) 37, no. 10, 785–791.

[34] Wang H. , Zhu L. S. , Pang C. J. , and Fan Q. , Repeatability Assessment of Anterior Segment Measurements in Myopic Patients Using an Anterior Segment Oct With Placido Corneal Topography and Agreement With a Swept-Source Oct, BMC Ophthalmology. (2024) 24, no. 1, 10.1186/s12886-024-03448-z. PMC11036772 38649848

[35] Hashemi H. , Miraftab M. , Panahi P. , and Asgari S. , Biometry and Intraocular Power Calculation Using a Swept-Source Optical Coherence Tomography: A Repeatability and Agreement Study, Indian Journal of Ophthalmology. (2022) 70, no. 8, 2845–2850, 10.4103/ijo.IJO_249_22. 35918927 PMC9672702

[36] Jeong J. , Song H. , Lee J. K. , Chuck R. S. , and Kwon J. W. , The Effect of Ocular Biometric Factors on the Accuracy of Various Iol Power Calculation Formulas, BMC Ophthalmology. (2017) 17, no. 1, 10.1186/s12886-017-0454-y, 2-s2.0-85018324899. PMC5414130 28464806

[37] Ji Z. , Ma J. , Sun Z. , and Zhang M. , Comparison of Two Formulas for Calculating Intraocular Lens in Patients With Angle-Closure Glaucoma, Medicine (Baltimore). (2024) 103, no. 45, 10.1097/md.0000000000040387. PMC11556955 39533616

[38] Che S. A. , Seong M. , Kim K. , and Lee Y. W. , Comparison of the Refractive Prediction Errors of Artificial Intelligence Formula With 3 Conventional Formulas and 11 Combination Methods in Cataract Surgery on Eyes With Short Axial Length, Arquivos Brasileiros de Oftalmologia. (2024) 88, no. 2, e20230215–e20230217, 10.5935/0004-2749.2023-0215. 39319909 PMC12668695

[39] Kim J. , Park J. , and Jo Y. , Comparison of the Formula Accuracy for Calculating Multifocal Intraocular Lens Power: A Single Center Retrospective Study in Korean Patients, Scientific Reports. (2024) 14, no. 1, 10.1038/s41598-024-54889-x. PMC10891126 38396107

[40] Xu H. , Zheng Y. , Lu X. et al., Accuracy of 7 Intraocular Lens Power Calculation Formulas in Primary Angle-Closure Glaucoma Eyes, According to Axial Length and Anterior Chamber Depth, BMC Ophthalmology. (2025) 25, no. 1, 10.1186/s12886-025-04238-x. PMC12306112 40721761

[41] Darcy K. , Gunn D. , Tavassoli S. , Sparrow J. , and Kane J. X. , Assessment of the Accuracy of New and Updated Intraocular Lens Power Calculation Formulas in 10 930 Eyes from the UK National Health Service, Journal of Cataract & Refractive Surgery. (2020) 46, no. 1, 2–7, 10.1016/j.jcrs.2019.08.014. 32050225

[42] Cooke D. L. and Cooke T. L. , Comparison of 9 Intraocular Lens Power Calculation Formulas, Journal of Cataract & Refractive Surgery. (2016) 42, no. 8, 1157–1164, 10.1016/j.jcrs.2016.06.029, 2-s2.0-84999019303. 27531292

[43] Kim J. W. , Eom Y. , Yoon E. G. et al., Algorithmic Intraocular Lens Power Calculation Formula Selection by Keratometry, Anterior Chamber Depth and Axial Length, Acta Ophthalmologica. (2022) 100, no. 3, e701–e709, 10.1111/aos.14956. 34378871 PMC9292369

[44] Yan C. and Yao K. , Effect of Lens Vault on the Accuracy of Intraocular Lens Calculation Formulas in Shallow Anterior Chamber Eyes, American Journal of Ophthalmology. (2022) 233, 57–67, 10.1016/j.ajo.2021.07.011. 34293335

[45] Huang G. , Gonzalez E. , Peng P. H. et al., Anterior Chamber Depth, Iridocorneal Angle Width, and Intraocular Pressure Changes After Phacoemulsification: Narrow vs Open Iridocorneal Angles, Archives of Ophthalmology. (2011) 129, no. 10, 1283–1290, 10.1001/archophthalmol.2011.272, 2-s2.0-80053907220. 21987670

[46] Bilak S. , Simsek A. , Capkin M. , Guler M. , and Bilgin B. , Biometric and Intraocular Pressure Change After Cataract Surgery, Optometry and Vision Science. (2015) 92, no. 4, 464–470, 10.1097/opx.0000000000000553, 2-s2.0-84926322029. 25785531

[47] Dong Z. , Hao J. , Wan Y. , and Wang N. , Influence of Shallow Anterior Chamber Depth on the Refractive Status After Phacoemulcification Combined With Intraocular Lens Implantation, Ophthalmology in China. (2017) 26, no. 6, 397–399.

[48] Yang F. , Hou X. , Wu H. , and Bao Y. , Analysis of Refractive Status After Cataract Surgery in Age-Related Cataract Patients With Shallow Anterior Chamber, Chinese Journal of Ophthalmology. (2014) 50, no. 2, 84–88. 24735660

[49] Sun J. , Feng Z. H. , and Xu H. , Analysis of the Refractive Status in Patients With Age-Related Cataract and Shallow Anterior Chamber After Phacoemulsification, International Eye Science. (2020) 20, no. 10, 1775–1779, 10.3980/j.issn.1672-5123.2020.10.24.

[50] Svoronos A. A. , O’Grady C. S. , Walker E. et al., Analysis of Spaceflight-Associated Biometric and Refractive Changes in Astronauts, American Journal of Ophthalmology. (2025) 276, 146–156, 10.1016/j.ajo.2025.04.001. 40194644 PMC12629133

[51] Kiristioglu M. O. , Doganay S. , and Ucan Gunduz G. , Axial Length and Iol Power Stability in Macular Edema Treated With Anti-Vegf: A Preliminary Study Using Olcr Biometry, BMC Ophthalmology. (2025) 25, no. 1, 10.1186/s12886-025-04188-4. PMC12220589 40597037

[52] Ning X. , Yang Y. , Yan H. , and Zhang J. , Anterior Chamber Depth-A Predictor of Refractive Outcomes After Age-Related Cataract Surgery, BMC Ophthalmology. (2019) 19, no. 1, 10.1186/s12886-019-1144-8, 2-s2.0-85068185898. PMC6591866 31238910

